# Frailty in community-dwelling older adults: reliability and validity of the Turkish version of the Gérontopôle frailty screening tool

**DOI:** 10.55730/1300-0144.5549

**Published:** 2022-06-26

**Authors:** Serdar CEYLAN, Merve GÜNER OYTUN, Arzu OKYAR BAŞ, Meltem KOCA, Burcu Balam DOĞU, Meltem Gülhan HALİL, Mustafa CANKURTARAN, Cafer BALCI

**Affiliations:** Division of Geriatrics, Department of Internal Medicine, Faculty of Medicine, Hacettepe University, Ankara, Türkiye

**Keywords:** Community, frailty, older adults, Turkish population

## Abstract

**Background/aim:**

Recognizing frailty had a great importance in countries with an increasing geriatric population. The study aims to evaluate the reliability and validation of the Gérontopôle frailty screening tool (GFST), which was developed to screen frailty in the community-dwelling older adults, for the Turkish population.

**Materials and methods:**

In this crosssectional study, participants who applied to the geriatrics outpatient clinic of a university hospital were included. Comprehensive geriatric assessments of all patients were performed. The validity of the GFST was tested by its concordance with the FRAIL scale. Test-retest and interrater reliability analyses were made.

**Results:**

Ninety-six patients were included in the study. Sixty-one and five-tenths percent (n = 59) of them are females. The median age was 72.0 (IQR: 10.0). There was a moderate concordance between the GFST and the FRAIL scale (Cohen’s kappa: 0.566, p < 0.001). The Gérontopôle frailty screening tool interrater and retest reliabilities were excellent (Cohen’s kappa: 0.814, p < 0.001 and 1.0, p < 0.001; respectively). The sensitivity of the GFST determined according to the FRAIL scale is 69.39%, the specificity is 86.36%, the positive predictive value is 85.00%, and the negative predictive value is 71.70%.

**Conclusion:**

The Gérontopôle frailty screening tool, which can be used by all healthcare professionals, is a valid and reliable tool for the Turkish geriatric population.

## 1. Introduction

Frailty is a clinical condition that decreases the response to stressors with the decline in physiological reserves and accordingly increases the predisposition to negative clinical outcomes [1, 2]. With the rise in life expectancy, the number of individuals living with frailty also increases [3]. According to the screening tool used, the prevalence of older adults living with frailty changes between 4.0% and 59.1% [4]. The increasing number of individuals living with frailty is becoming an important public health problem [5]. Along with increasing complicated health conditions, there is an increase in health expenditures. For these reasons, identifying the patient living with frailty and taking precautions are essential [6]. Physicians, who provide primary health care services, are the first and most frequently encountered physician group for the patients. It is important for these physicians to recognize patients living with frailty and refer them to a frailty center after implementing appropriate interventions [7].

Many frailty screening tools have been developed and there is no gold standard tool to identify frailty [8]. Many of these are complex to implement in primary health care. The frailty screening tools to be used in primary health care should be as reliable and simple as possible to detect frailty [9]. The Gérontopôle frailty screening tool (GFST) is one of the tools developed following these criteria [10]. The Gérontopôle frailty screening tool, which was developed by the Gérontopôle of the Toulouse University Hospital, has been made available to general practitioners. After the comprehensive geriatric assessment, necessary interventions are made to the patients for frailty. Ninety-five and two-tenths percent of patients referred by general practitioners using GFST were found to be prefrail or frail according to the FRIED frailty phenotype [10].

Recognizing frailty is of paramount importance in countries with an increasing geriatric population. There is a need for easy-to-apply, valid and reliable screening tools to facilitate the recognition of frailty. The study aims to evaluate the reliability and validation of the GFST in the Turkish population.

## 2. Materials and methods

### 2.1. Participants

Patients who were admitted to the geriatric outpatient clinic of a university hospital between 01.12.2021 and 31.01.2022 were included in the study. Inclusion criteria for the study were 65 years and older, consent to be included in the study, Katz index of independence in activities of daily living score (ADL) ≥5, and no acute illness. Demographic characteristics, chronic diseases, and medications were recorded. Within the scope of the comprehensive geriatric evaluation, Katz ADL [11], Lawton-Brody instrumental activities of daily living scale [12], mininutritional assessment-short form [13], minimental state examination [14], geriatric depression scale-15 [15], timed up and go test [16], SARC-F [17], 4-meter gait speed test^1^ and grip strength^2^ measurement were performed. The medical, social, psychological, and cognitive information required for frailty screening scales were obtained. Multimorbidity was defined as the presence of ≥2 chronic diseases [18].

### 2.2. Study tool

The Gérontopôle frailty screening tool is a scale developed for the use of general practitioners to screen frailty in individuals aged 65 and over, without the presence of acute disease and disability [19]. The Gérontopôle frailty screening tool consists of three stages. In the first stage, patients are evaluated for Katz ADL. Those who score less than five do not need to proceed to the questionnaire stage. In the second stage, there are six questions to be answered by the physician about the patient. These questions are “does your patient live alone, has your patient involuntarily lost weight in the last 3 months, has your patient been more fatigued in the last three months, has your patient experienced increased mobility difficulties in the last three months, has your patient complained of memory problems, does your patient present slow gait speed (i.e. >4 s to walk 4 m). The answer options are yes, no, or do not know. When at least one of these questions is answered “yes”, the third stage of the test is passed. At this stage, there is the question(s) to be answered by the physician again. They are answered with yes or no. First, “do you think your patient is frail” question needs to be answered. If this question is answered “yes”, the second question is “is your patient willing to be assessed for his/her frailty status at a future frailty clinic”. The test is ended by answering this question as yes or no. Some patients were retested for reliability by performing a second evaluation by the same physician two weeks after the initial evaluation with the GFST. In addition, some patients were evaluated by a physician in another room to test their interrater reliability.

### 2.3. Translation

The translation and cultural adaptation were made according to the recommendations of the ISPOR Task Force [20]. To validate the language, first of all, the GFST was translated from English to Turkish by native Turkish-speaking physicians who are experts in translation and can speak fluent English. All authors agreed on the Turkish translation. After the translation control was done, the Turkish version of the test was translated back into English by two native English speaker academicians who did not know the original. Thus, language validation was performed with the “forward-backward translation” method. Finally, the test was administered to older adults living in the community by physicians to assess the cultural adaptation.

### 2.4. Reference tools

The reference frailty scale was the FRAIL scale which consists of 5 domains. It was developed by the Geriatric Advisory Panel of the International Academy of Nutrition and Aging as a frailty scale that is easy to use, takes minimum duration, and can be used by all healthcare professionals. It can be completed without the need for any tools or tests. As a result of the questioning of fatigue, resistance, ambulation, illnesses, and loss of weight, a decision is made about the frailty of the patient. For fatigue, “how much of the time during the past 4 weeks did you feel tired” question is asked to the patient. One of the following answers is requested: “1 = all of the time, 2 = most of the time, 3 = some of the time, 4 = a little of the time, 5 = none of the time”. Those who choose answers 1 or 2 get 1 point. Other options are 0 points. For resistance, a “yes” or “no” answer is required to the question “by yourself and not using aids, do you have any difficulty walking up 10 steps without resting”. Answering “yes” to these two questions is 1 point. To learn about illnesses, the patient is asked whether he has “hypertension, diabetes, cancer (other than a minor skin cancer), chronic lung disease, heart attack, congestive heart failure, angina, asthma, arthritis, stroke, and kidney disease”. If he has five or more illnesses, he gets 1 point. For weight loss, the current weight of the patient is compared with the weight 1 year ago. The percentage of weight loss, if any, is calculated. More than 5% weight loss is 1 point. Zero points are evaluated as robust, 1–2 points as prefrail, and 3 or more points as frail [21]. Turkish reliability and validity were done by Hymabaccus et al. in 2017 [22].

Katz ADL is a tool that consists of 6 items and is used to evaluate the disability of the patient. Items are bathing, mobility, eating, dressing, continence, and toileting. Four or fewer points represent physical disability [23]. Validity and reliability for Turkish older adults have been proven by the study conducted by Arık et al. [11].

### 2.5. Ethical approval

The study protocol was approved by the local ethics committee of Hacettepe University Faculty of Medicine (project number: GO/21/1312, decision number: 2022/02-39).

### 2.6. Statistical analysis

The sample size was calculated using the two rater kappa statistics [24] by providing 90% power to determine the correct kappa when two categories according to the FRAIL scale robust and prefrail/frail frequencies in Turkey [22] were 0.42 and 0.58, respectively. The significance value was accepted as 0.05.

Statistical analysis was performed using SPSS 24.0. Categorical variables were expressed as numbers and percentages, numerical variables were expressed as mean and standard deviation or median and interquartile range according to the normal distribution situation. To evaluate the construct validity of the GFST, the FRAIL scale was accepted as the reference tool. Cohen’s kappa was used to evaluate the assessment agreement between robust and frail categories. The FRAIL scale was classified as robust and prefrail/frail when looking at its concordance with the GFST. Cohen’s kappa was also used to investigate interrater and retest reliabilities. Sensitivity, selectivity, positive and negative predictive values were calculated. p-value of <0.05 was accepted as statistically significant.

## 3. Results

Ninety-six patients were included in the study. Sixty-one and five-tenths percent (n = 59) of them were female. The median age was 72.0 (IQR: 10.0). While 61 (63.5%) patients were married, 22 (22.9%) patients were illiterate. The mean body mass index was 29.19 ± 5.76. The number of patients with multimorbidity was 67 (69.8%). The most common geriatric syndromes were polypharmacy with a prevalence of 52.1% (n = 50) and urinary incontinence with a prevalence of 40.6% (n = 39). The median score of the FRAIL scale is 1.0 (IQR: 2.0). In the classification made according to the score obtained, 44 patients (45.8%) were robust, 29 patients were prefrail (30.2%) and 23 patients (24.0%) were frail. Characteristics, chronic diseases, comprehensive geriatric assessment results, and frailty status were given in Table 1.

When we evaluated the concordance of the GFST and the FRAIL scale, there was a moderate concordance (Cohen’s kappa: 0.566, p < 0.001). The GFST interrater and retest reliabilities were excellent (Cohen’s kappa: 0.814, p < 0.001 and 1.0, p < 0.001, respectively) (Table 2).

The sensitivity of the GFST determined according to the reference scale was 69.39%, the specificity was 86.36%, the positive predictive value was 85.00%, and the negative predictive value was 71.70%. When we examine the likelihood ratios, it was calculated as 5.55 for positive and 0.35 for negative.

## 4. Discussion

The study was conducted to demonstrate the validity and reliability of the Gérontopôle frailty screening tool in the Turkish geriatric population. As a result of the evaluation made by taking the FRAIL scale as a reference, there was a moderate concordance between the GFST and the FRAIL scale in frailty assessment. It has also a good specificity. Therefore, it has been shown that the GFST is a valid and reliable frailty screening tool in the older adults of Turkey.

Living with frailty risk increases with advancing age. As a result of a review study, the prevalence of frailty was 10.7% in community-dwelling older adults. [4]. This prevalence rate rises with advancing age [25]. The geriatric population in the world is climbing.^3^ As the geriatric population increases, the truth emerges that the number of individuals living with frailty rises. This rise leads to an increase in the number of individuals in need of care and increases health expenditures [26]. Mortality, hospitalization, number of hospital admissions, prolonged length of hospitalization, decrease in quality of life, falls are more common in patients living with frailty [25, 27–29]. With early recognition of frailty and taking necessary precautions, these adverse health outcomes can be reduced [30].

Comprehensive geriatric assessment is the best method for evaluating older adult patients. With the comprehensive geriatric evaluation; for improving health outcomes of the patient; functioning, physical health, cognition, mood, nutritional status, balance, gait speed, grip strength, medications, bone mineral density, fall risk, socioeconomic circumstance, chronic diseases of the patient are examined [31]. Comprehensive geriatric assessment is of great importance for detecting frailty and making a care plan. However, CGA is a practice that takes a long time, which prevents it from being applied by most physicians [32]. Therefore, practical, easy, and quick to apply scales have been developed. One of these is the GFST developed by the geriatric assessment center called Gérontopôle of Toulouse in France [10]. After the questions are in the form of a questionnaire without acute disease and nondependent patients, frailty determination is made by the physician’s clinical decision who made the evaluation. Besides geriatricians, it can be used by general practitioners and other health professionals because it does not require a special device, can be done in a short time and easily [33]. In addition, another positive aspect of the GFST is that it does not look at frailty from just one perspective, also guides physicians in terms of social, physical, and cognitive frailty.

It is of great importance that this frailty screening scale is used by general practitioners. To recognize community-dwelling frail individuals, general practitioners, who are the physicians who encounter the geriatric population the most, need to be aware of frailty. The GFST appears to be an appropriate tool for GPs and other health professionals to recognize frailty early and guide the patient for further evaluation. Thus, it will be possible to reduce the negative health consequences related to frailty. Validation of the GFST in more languages will serve this purpose. So far, there are versions in seven languages.^4^ As a result of our study, Turkish validity and reliability were demonstrated.

Turkey is a developing country where the geriatric population is increasing and will soon be among the aged countries. In the 2021 data of Turkish Statistical Institute, the number of individuals aged 65 and over has increased to 8,245,124, and the ratio has increased to 9.7%.^5^ This ratio is expected to be 12.9% in 2030.^6^ Two studies with high patient numbers evaluate the frequency of frailty in Turkey. In the study conducted by Eyigör et al. using the FRIED frailty index, the prevalence of frail patients was 39.2% [34]. The other study was done by Akın et al. that the Fried frailty index and the FRAIL scale were used. The frequency of frail participants is 27.8% according to FFI and 10.0% according to the FRAIL scale [35]. There will inevitably be an increase in the number of frail individuals with the prolongation of life expectancy, conditions such as high illiteracy rate, low socioeconomic level, and rise in multimorbidities in Turkey. That is why frailty scales that can be used widely should be validated in the Turkish population to recognize frailty and intervene early. Validation of the GFST, which can be used by all healthcare professionals, has a great significance in this respect.

As a result of this study, it has been shown that the GFST, which can be used by all healthcare professionals, is a valid and reliable tool for the Turkish geriatric population. With the widespread use of the GFST by health professionals, adverse health outcomes related to frailty can be reduced.

## Figures and Tables

**Table 1:** Demographic and clinical characteristics of patients.

	N = 96 (n, %)
Characteristics	
Age (median, IQR)	72.0 (10.0)
Sex (female)	59 (61.5)
Illiterate	22 (22.9)
Marital Status (married)	61 (63.5)
Body mass index (mean, SD)	29.19 ± 5.76
Smoking	36 (37.5)
Multimorbidity ≥2	67 (69.8)
Comprehensive geriatric assessment-geriatric syndromes	
Dementia	4 (4.2)
Depression	27 (28.1)
Osteoporosis	21 (21.9)
Falls	20 (20.8)
Polypharmacy	50 (52.1)
Drug number (median, IQR)	5.0 (4.0)
Urinary incontinence	39 (40.6)
Katz index of independence in activities of daily living (median, IQR)	6.0 (1.0)
Lawton-Brody instrumental activities of daily living scale (median, IQR)	8.0 (0.0)
Mininutritional assessment-short form (median, IQR)	13.0 (4.0)
Minimental state exam (median, IQR)	28.0 (5.0)
Yesavage geriatric depression scale (median, IQR)	2.0 (6.0)
SARC-F (median, IQR)	1.0 (3.0)
Grip strength(mean, SD)	Females: 17.73 ± 5.02 Males: 27.39 ± 7.41
Gait speed (m/s) (mean, SD)	0.95 ± 0.52

N: number, IQR: interquartile range, SD: standard deviation, m: meter, s: second.

**Table 2:** Gérontopôle frailty screening tool and reference test concordance results.

		Gérontopôle frailty screening tool		Kappa	p
		Robust	Frail		
The FRAIL scale				0.566	<0.001
	Robust	38 (71.7)	6 (14.0)		
	Prefrail/frail	15 (28.3)	34 (86.0)		
Interrater reliability		-	-	0.814	<0.001
Retest reliability		-	-	1.0	<0.001

## Footnotes

1 Comprehensive Geriatric Assessment Toolkit. Gait Speed Test. Resources for the Comprehensive Geriatric Assessment based Proactive and Personalised Primary Care of the Elderly [online]. Website: https://www.cgakit.com/fr-1-gait-speed-test [accessed 31 December 2021].

2 Comprehensive Geriatric Assessment Toolkit [Internet]. Grip Strength. Resources for the Comprehensive Geriatric Assessment based Proactive and Personalised Primary Care of the Elderly [online]. Website: https://www.cgakit.com/grip-strength [accessed 31 December 2021].

3 United Nations. Population Division World Population Prospects 2019 [online]. Website: https://population.un.org/wpp/Download/Standard/Population/ [accessed 31 December 2021].

4 Frailty.net Diagnostic Tools [online]. Website: https://frailty.net/ [accessed 31 December 2021].

5 Turkish Statistical Institute. The Results of Address Based Population Registration System (2021) [online]. Website: https://data.tuik.gov.tr/Bulten/Index?p=The-Results-of-Address-Based-Population-Registration-System-2021-45500 [accessed 31 December 2021].

6 Turkish Statistical Institute. Elderly Statistics (2020) [online]. Website: https://data.tuik.gov.tr/Bulten/Index?p=Elderly-Statistics-2020-37227 [accessed 31 December 2021].
